# Dorsal onlay buccal mucosa graft urethroplasty for bulbar urethral stricture: a single centre experience

**DOI:** 10.11604/pamj.2020.36.305.21398

**Published:** 2020-08-19

**Authors:** Idorenyin Cletus Akpayak, Samaila Ibrahim Shuaibu, Chima Gideon Ofoha, Ayodele Olufikayo Oshagbemi, Nuhu Kutan Dakum, Venyir Mamzhi Ramyil

**Affiliations:** 1 Urology Division, Surgery Department, Jos University Teaching Hospital, Jos, Nigeria

**Keywords:** Urethral structure, buccal mucosal graft, urethroplasty

## Abstract

**Introduction:**

the successful treatment for urethral strictures demands not just attention to surgical details but careful selection of the reconstructive technique. For long segment urethral strictures substitution urethroplasty is required. This study sought to determine the success rate and complications of dorsal onlay buccal mucosal graft (BMG) urethroplasty for long segment urethral strictures in our hospital.

**Methods:**

this was a retrospective study carried out at Jos University Teaching Hospital from March 2015 to March 2018. The case notes of male patients who had dorsal onlay buccal mucosal graft urethroplasty for long segment bulbar urethral stricture within the study period were retrieved. Patients´ demographics, cause and nature of urethral strictures, duration of follow up, the success rate and complications were collected and subjected to statistical analysis using SPSS® version 22.

**Results:**

twenty-four men with mean age of 45 years (range 14-67 years) had dorsal onlay buccal mucosal graft urethroplasty during the study period. The mean stricture length was 4.5cm (range, 2-7cm). After a mean follow up duration of 2 years (range, 1 4 years), 21(87.5%) patients had a successful urethroplasty as they were able to pass urine at one year post urethroplasty without lower urinary tract symptoms, while 3(12.5%) had recurrence of the urethral stricture. At the recipient site, 2(8.3%) patients had primary bleeding that did not require blood transfusion. Also, 2(8.3%) patients had superficial wound infection which was treated with antibiotics. At the donor site, 4(16.7%), 2(8.3%), 4(16.7%) had donor site swelling, transient bleeding and soreness respectively.

**Conclusion:**

dorsal onlay BMG urethroplasty has a good success rate and minor complications and therefore suitable for long segment bulbar urethral strictures.

## Introduction

The successful management of urethral strictures demands not only attention to surgical detail but careful selection of the reconstruction technique [1]. Urethral stricture greater than 2cm in length often cannot be repaired using end to end anastomosis and therefore require substitution urethroplasty [2]. Split and full thickness skin grafts, bladder mucosa and buccal mucosa have all been used [3]. In 1894, Sapezhko, a surgeon from Kiev, Ukraine was the first to fully describe the use of oral mucosa from the lip and mouth in 4 patients requiring urethral surgery [4]. Humby, in 1941 then explored the use of buccal mucosa graft (BMG) for hypospadias repair [5]. Then El-kasaby [6] and colleagues reported the use of oral mucosa from the lip as a free graft for the management of both penile and bulbar strictures. Morey [7] and colleague in 1996 described the ventral onlay technique while Barbagli [8] and colleagues established the use of dorsal onlay technique for the BMG urethroplasty. Buccal mucosal graft is an excellent urethral substitute because of ease of harvest, surgical handling characteristics, hairlessness, compatibility in a wet environment, and its early ingrowth and graft survival [9]. It is less prone to stricture recurrence, the thick buccal mucosa epithelium and dense submucosa and extensive capillary network assure rapid neovascularization and early access to nutrients from the wound bed [9,10].

Buccal mucosa graft can be placed laterally for bulbar urethral reconstruction; however, placing the graft dorsally or the ventrally is the most widely practiced technique [11]. There is a debate as to whether BMG should be placed dorsally or ventrally for bulbar urethral reconstruction; however, to date, there is no clear winner or loser [11-13]. Dorsal onlay grafting may seem more technically difficult and sometimes more aggressive procedure but it could be applied in the reconstruction in all parts of the bulbar urethra. On the other hand, the application of the ventral onlay technique to the distal bulbar urethral reconstruction is not satisfactory because of the insufficient spongiosum support. This lack of mechanical support to the graft and in turn, vascular supply leads to a higher risk of urethrocutaneous fistula, pseudodiverticulum causing postvoid dribbling and ejaculatory dysfunctions [8]. This study was carried out to report our experience with dorsal onlay BMG urethroplasty for long segment urethral strictures at our hospital and specifically to determine the success rate and the complications of the dorsal onlay BMG urethroplasty.

## Methods

This is a retrospective study of male patients who had dorsal onlay buccal mucosal graft urethroplasty (BMG) for bulbar urethral strictures between 2015 and 2018 at Jos University Teaching Hospital. Records of 24 patients who had the surgery were reviewed and included in the study. All patients included in the study had a thorough evaluation. The evaluation was aimed at detecting the etiological factors such as poorly treated urethritis, trauma, instrumentation, or prolonged urethral catheterization. All patients included in the review had either retrograde urethrogram (RUG) or micturating cystourethrogram (MCUG) or both to determine the length, site and multiplicity of the stricture. Patients who had ventral onlay BMG urethroplasty or ´double-faced´ BMG urethroplasty for nearly obliterative strictures were excluded from the study. Also, patients who had staged urethroplasty with BMG were excluded. The patients used mouthwash containing chlorhexidine a day before surgery. Details of their biodata, clinical presentation and cause of the urethral stricture, investigations, operative treatment, postoperative complications and other outcomes of surgery were extracted. Data were entered into and analysed using SPSS® version 22.

**Figure 1:**
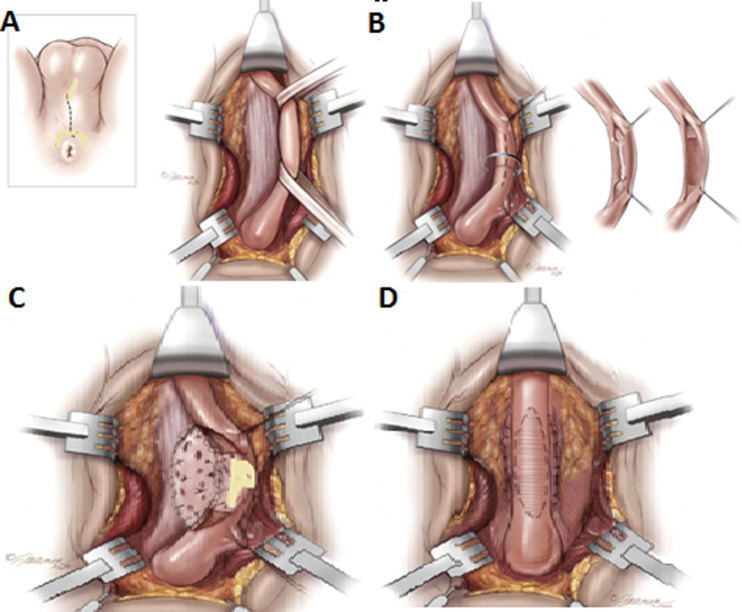
the steps of the dorsal onaly buccal mucosa graft urethroplasty. A) midline perineal incision; B) mobilized bulbar urethra and dorsal urethrotomy; C) buccal mucosa quilted to the tunica albuginea; D) completed buccal mucosa grafting in dorsal onlay fashion

**Surgical technique:** patients had general anaesthesia with either nasal or oropharyngeal/endotracheal intubation with the patient placed in lithotomy position. A midline incision was also made on the skin and deepened to expose the corpus spongiosus muscle. The corpus spongiosus muscle was dissected off the underlying urethra and the retractor repositioned deeply. A dorsal midline stricturotomy was carried out and adequate haemostasis achieved (Figure 1) [14]. The same team was always responsible for the harvest of the buccal mucosa. This required gloves change and a different set of relevant instruments. The patient face was draped with mouth exposed after routine skin preparation. The mouth cavity was widely opened using a mouth retractor. An indelible marker was used to outline the margin of the intended graft. Infiltration of the submucosal tissue with 1: 100,000 adrenaline allowed the mucosa to be dissected off the buccal muscles with ease and reduce bleeding. The raw area was packed with wet gauze. We took rectangular-shaped grafts and did not close the donor sites. The graft was defatted and quilted to the corpora carvanosa. Then, urethral was closed on the buccal mucosa graft over a size 16Fr silicone urethral catheter. This was followed by reconstitution of the corpus spongiosus. The corpus spongiosus muscle was meticulously re-apposed using vicryl 3/0. The remaining wound was closed in three layers with vicryl 2/0 and covered with a firm occlusive dressing. The urethral catheter was removed after 4 weeks. Follow-up was for 1 to 4 years, with a mean follow-up duration of 2 years. In this study, success was defined as the ability of patients to pass urine satisfactorily without lower urinary tract symptoms (LUTS).

## Results

A total of 24 patients had dorsal buccal mucosa graft urethroplasty within the study period. Their mean age was 45 years (range 14-67). The age group 51-60 years was the most affected. The aetiologies of the urethral stricture were infection, which accounted for 70.8% of the causes, trauma 16.7, Idiopathic 8.3% and toxic catheter with 4.2% (Table 1). The mean urethral stricture length was 4.5cm (range 2-7cm). Fifteen patients (62.5%) had suprapubic cystostomy (SPC) before urethroplasty due to either acute or chronic urinary retention, while 9(37.5%) had no SPC. Out of the 24 patients, 22(91.7%) had no previous surgeries or intervention. One had urethral dilatation and 1 had anastomotic urethroplasty previously. None of the patients had blood transfusion. All the patients had general anaesthesia with nasal intubation in 8(33.3%) cases and oropharyngeal/endotracheal intubation in 16(66.7%). The mean intra-operative time was 2.5 hours, while the mean hospital stay was 4.5 days. Twenty-one (87.5%) of the patients had their urethral catheter removed in 4 weeks, while 3(12.5%) patients had their removed in 5 weeks. After a mean follow up period of 2 years, 21(87.5%) patients had a satisfactory urinary stream (Table 2).

**Table 1:** causes of urethral stricture in the 24 patients

Aetiology	Frequency	Percentage (%)
Infection	17	70.8
Trauma	4	16.7
Idiopathic	2	8.3
Toxic catheter	1	4.2
Total	24	100

**Table 2:** success rate and complications after dorsal onlay buccal mucosal graft bulbar stricture urethroplasty in the 24 patients

Complications	Number of patients, n=24	Percentage (%)
**Donor site**		
Oedema/swelling	4	16.7
Bleeding	2	8.3
Soreness/restriction of the mouth opening	4	16.7
Stentions duct injury/stenosis	0	0
**Complications**		
**Recipient site**		
Superficial wound infection	2	8.3
Erectile dysfunction	0	0
Bleeding	2	8.3
**Duration of follow up**		
4years	10	41.7
<4years	14	58.3
**Success at 2 years**		
No recurrence	21	87.5
Recurrence	3	12.5

## Discussion

Our study revealed a success rate of 87.5%. This is within the reported success rate of dorsal onlay BMG urethroplasty by many authors which is between 85% to 95% [12,15-18]. The wide variation noticed in the reports of different authors has been attributed to the difference in the duration of follow up, the definition of surgical success (recurrence), previous treatments and the aetiology of the strictures. While the current study defined recurrence as resurgence in lower urinary tract symptoms (LUTS) other authors have reported the same as uroflowmetry <15ml/s and/or imaging showing a significant narrowing of the urethral lumen [19]. Yet others define recurrence disease as the need for secondary intervention or failure to pass a size 16Fr urethral catheter at urethroscopic assessment [11,20,21]. For report of success rate, longer follow up has been advocated. For instance, Barbagli has suggested that stricture should be classified as cured only after 6 years of follow up [22]. However, it is difficult to discard shorter term reports as most (75%) of the stricture recurrence occur within the first 6 months after surgery [20]. We observed complications superficial wound infection, bleeding that did not require blood transfusion. We did not, however, observe urethrocutaneous fistula or complaints of erectile dysfunction. Barbagli [8] and colleagues have worried that aggressive urethral dissection during dorsal onlay BMG urethroplasty could probably damage the cavernosal nerves. For example, Coursey [23] has reported 19.2% cases of ED with BMG urethroplasty. However, many others did not find this complication in their series, including prospective studies demonstrating no difference in the international index of erection function (IIEF) [24,25]. Therefore, despite the explanation by Barbagli [8] and colleagues, the effect of dorsal onlay BMG urethroplasty of erectile function remains a debate.

Many authors have found that donor site complications have low morbidity [26-28]. Equally, our study recorded few and transient complications at the donor site, which included swelling, bleeding and soreness. Two (8.3%) of our patients had transient bleeding, which was easily controlled by leaving a gauze ball in the cheek for 4 hours. Kane [29] and colleagues noted this complication in one patient in their review of donor site complications in 53 patients confirming that this is not a common complication. Bleeding in their patients required repeated evacuation and was attributed to thick harvest with significant injury to the buccinators muscle. We recorded mild soreness, swelling and restriction of the mouth opening in 4 (16.7%) of our patients and they subsided in a few days after surgery. In our study, we attributed the restriction of the mouth to the soreness and the swelling. Restriction of the mouth opening occurs more frequently and is prolonged where the donor site is closed. For instance, Dublin [30] and colleagues in their assessment of oral complications of BMG harvest in 35 patients found restriction of mouth opening in 38% of their patients and most did not resolve after 3 weeks. In the current study, we did not observe complications like numbness as has been reported by other authors. Kamp [31] and colleagues compared donor site complications from inner check to that of lower lip also found none of their 24 patients had numbness or other sensory neural deficits in the BMG group. They suggested that trauma to the buccal nerves during BMG harvesting is of less clinical significance when compared to trauma to mental nerve during harvesting from the lip. They recommended that BMG harvesting whenever possible for urethral reconstruction. This study is limited by its retrospective nature, the small number of patients and short follow up duration, which has affected a more satisfactory conclusion. Our success was also based on patient-reported absence of LUTS as recording of postoperative maximum flow and postoperative retrograde urethrogram were incomplete and could not be reported.

## Conclusion

Dorsal onlay buccal mucosal graft urethroplasty is considered a safe and suitable technique for long segment bulbar urethral stricture.

### What is known about this topic

- Long segment bulbar urethral stricture requires substitution urethroplasty;
- Different substitution urethroplasty abound with report of different success rates in literature;
- There are complications of substitution urethroplasty other than recurrence.

### What this study adds

- Success rate of dorsal onlay buccal mucosa graft urethroplasty in our patients;
- The complications found after dorsal onlay buccal mucosa graft urethroplasty in our patients;
- Dorsal onlay buccal mucosa graft urethroplasty is a versatile technique for long segment bulbar urethral stricture.
